# A novel multiple plant‐based milk alternative containing various preprocessed grains achieves better performance in protein digestibility and free amino acid profile via in vitro gastrointestinal digestion analysis

**DOI:** 10.1002/fsn3.4177

**Published:** 2024-06-26

**Authors:** Xue Wang, Lu Zhang, Mohan Wang, Hongjiang Ma, Shiwei Liu, Meng Wang, Youqiang Yu, Guoyu Liu, Qiuge Cao, Xi Wang, Xishan Ma, Peng Yuan, Jia Liu, Yongjiu Zhang, Shenglin Duan

**Affiliations:** ^1^ Heilongjiang Feihe Dariy Co., Ltd. Beijing China; ^2^ Heilongjiang Beiwei 47 Plant Protein Co., Ltd. Heilongjiang China; ^3^ Beijing Key Laboratory of the Innovative Development of Functional Staple and Nutritional Intervention for Chronic Diseases China National Research Institute of Food and Fermentation Industries Co., Ltd. Beijing China

**Keywords:** fermented cereals, germinated soybeans, in vitro gastrointestinal digestion, plant‐based milk, protein digestion

## Abstract

Plant‐based milk alternatives are sustainable, hypoallergenic, and nutrient‐rich, but challenges related to their lower bioavailability compared with animal‐based milk still exist. In this study, we developed a multiple plant‐based milk alternative using germinated soybeans and fermented cereals, and compared the protein digestible behaviors with commercial soy and bovine milk via in vitro gastrointestinal digestion. The multiple plant‐based milk alternative possessed a higher level of essential amino acids and amino acid scores than the soy milk and a smaller percentage of low‐molecular‐weight peptides than the bovine milk. It displayed better protein‐digestible responses with no apparent gastric coagulation. Moreover, the relatively larger particles in the multiple plant‐based milk alternative had few effects on protein digestibility, with the highest proteolytic degree and a better free amino acid profile. The findings suggest that the multiple plant‐based milk alternative presents higher protein digestibility behavior, and it could be a promising industrial plant‐based product.

## Graphical abstract

Germination and fermentation as plant preprocessing techniques could be effective in improving the nutritional quality of plant‐based products. The multiple plant‐based milk alternative we developed possessed a higher proteolytic degree and better balanced amino acid profiles under in vitro digestion, suggesting that it would be a promising industrial plant‐based product with excellent nutritional value.

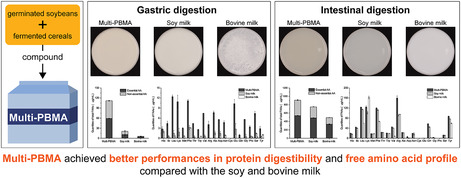

## 1 INTRODUCTION

In terms of protein‐rich foods, a growing awareness and emphasis will be placed on plant and animal protein. As is well known, animal protein plays an important role in providing the high‐quality essential nutrients in our daily food intake (Xiao et al., 2023). However, with the evolving consideration of future‐oriented social issues, protein consumption, tending toward differentiation, individualization, and simplification, has changed toward plant proteins (He et al., 2020). The intake of animal protein is often accompanied by a high intake of saturated fat, which possesses potential risks for chronic diseases such as hypertension and hyperlipidemia (Xiao et al., 2023). However, plant protein has the advantages of easy digestion and absorption and possesses a variety of physiological health functions (de Las Heras‐Delgado et al., 2023). Generally, plant‐based industry can not only reduce the raising and slaughter of livestock, land resource occupation, carbon emissions, and water consumption but also contribute to environmental sustainability and resource conservation (Lauk et al., 2022). Nowadays, compared with bovine milk, the popularity of plant‐based (PB) milk alternatives is increasing due to lactose‐free, hypoallergenicity, lifestyle choices (e.g. veganism), a more sustainable protein source, less ethical concerns about livestock animals, and so on (Ozturk & Hamaker, 2023). The variety and consumption demand of PB milk alternatives are gradually increasing as a result (Jeske et al., 2018). Nowadays, ‘plant‐based’ has represented one of the top three ‘global food, nutrition, and health trends’ in modern society, together with more beneficial grains and digestive wellness diversification (Guevara‐Zambrano et al., 2023).

It is known that PB milk alternatives are processed typically from one of the raw plant materials, such as soy, rice, almonds, oats, legumes, grains, nuts, and coconuts (Drewnowski, 2022; Geburt et al., 2022). And among a wide range of PB milk alternatives around the world, soy‐based milk alternatives are still the leading category, with almond, coconut, or oat‐based items also being focused on (Jeske et al., 2018). However, one commercially successful PB milk alternative needs to meet the substituted demand for the protein quality in bovine milk, which is generally regarded as a fundamental protein source for human growth and development (Antunes et al., 2022). Therefore, the mixture composition of various grains (e.g. soy, rice, and wheat) has been thoroughly investigated for enhancing the plant protein nutritional quality rather than a single plant protein source (Alrosan et al., 2022).

The nutritional quality of proteins in diets directly depends on their amino acid (AA) composition and bioavailability (Arranz et al., 2023). Compared to animal proteins, plant proteins have fewer essential AAs indispensable for human nutritional needs and more trypsin inhibitors, such as fiber, saponins, tannins, and other anti‐nutritional factors (Reynaud et al., 2020). Meanwhile, plant proteins are generally thought to be resistant to proteolysis, attributing to the significant disulfide bonds, hydrophobic interactions, and self‐association capacity in their cell wall composition and architecture (Capuano & Pellegrini, 2019; Carbonaro et al., 2015). Interestingly, new processing techniques, including germination, fermentation, heat treatment, and exogenous phytase, could not only reduce the anti‐nutritional factors but also enhance the digestibility and nutritional properties of plant proteins (Santos‐Hernandez et al., 2020; Vogelsang‐O'Dwyer et al., 2021). Specifically, germination that occurs naturally can advance the enzymatic degradation of plant proteins and their anti‐nutritional factors (saponins and trypsin inhibitors) (Singh et al., 2017). And fermentation enhances the digestibility, bioavailability, and bioaccessibility of plant proteins by disrupting complex protein cross‐linking via the enzymes produced by microorganisms (Alrosan et al., 2022). Therefore, germination and fermentation as plant preprocessing techniques are effective and available for improving the quality of PB milk alternatives. Many studies have focused on the technique of processing optimization or development. However, little research is available regarding the protein's digestive ability when different plant proteins are mixed in terms of digestibility, digesta features, and nitrogen fraction profiles.

The purpose of this study is to evaluate the protein digestible behavior between the multiple PB milk alternative (Multi‐PBMA) containing germinated soybeans and fermented cereals, commercial soy, and bovine milk, using a standardized in vitro gastrointestinal digestion system (INFOGEST 2.0). Protein and AA content, proteolytic degree, coagulation characteristics, particle size distribution, molecular weight distribution, and free amino acid (FAA) profiles were determined. It will provide valuable information on the gastrointestinal fate of complex plant proteins and promote the development of new PB milk alternatives as promising industrial plant‐based products.

## 2 MATERIALS AND METHODS

### 2.1 Materials

The porcine gastric mucosal pepsin (535 U/mg), porcine pancreatic pancreatin (4 × USP specifications), porcine pancreatic lipase (332 U/mg), and ninhydrin reagent (2% solution) were purchased from Sigma‐Aldrich (St. Louis, MO, USA). Porcine bile was purchased from Macklin Biotech Inc. (Shanghai, China). Standards of L‐leucine, Cytochrome C, porcine pancreatic insulin, bacitracin, glycyl‐glycyl‐tyrosyl‐arginine, and triglycine were purchased from Aladdin Reagent (Shanghai, China). Unless indicated otherwise, all other chemicals were obtained from China. High‐grade deionized water was used (>18.2 mΩ) throughout the study.

Multi‐PBMA preparation: The Dongsheng soybean was cleaned to remove impurities, soaked in 25°C water for 4 h, then taken out and germinated at 25°C for 18 h. The multiple cereals (the brown rice and white rice) were cleaned and soaked in water at 25°C for 10 h, and then taken out and steamed at 100°C for 1 h. After cooling to room temperature, 0.5% rice leaven (AngelYeast, Yichang, China) was added and mixed well into multiple cereals for fermenting 48 h at 30 ± 1°C. After the end, the fermented cereals were steamed at 100°C to sterilize for 30 min. The Multi‐PBMA ingredients, consisting of germinated soybeans combined with multiple fermenting cereals, were ground using a colloid mill (Shenyang Aerospace Xinguang Group, Shenyang, China) and modulated. Twofold homogenization was performed at 40 MPa using a high‐pressure homogenizer (NS2002H, GEA Engineering Technology, Beijing, China) to obtain a pre‐emulsion, which was subjected to ultrahigh‐temperature sterilization (HP‐DSI‐30, Shanghai, China) at 142°C for 4 s. The soy milk was prepared from the ungerminated Dongsheng soybean with the same grinding, homogenization, and sterilization process as the Multi‐PBMA. The bovine milk was commercially available and had been homogenized and sterilized. In addition, none of the food additives were added to all three samples.

### 2.2 Determination of nitrogen content and total AA profiles

The nitrogen content was determined using the Kjeldahl method. All samples were analyzed in triplicate.

The AA profiles were determined using HPLC‐MS/MS (Shimadzu LC‐20 AD, Shimadzu Corporation, Kyoto, Japan; API 3200MD TRAP, AB Sciex LLC, Framingham, Massachusetts, USA). Typically, two aliquots of each sample were digested at 110°C for 24 h, using LiOH (4 M) for tryptophan and HCl (6 M) for other AAs in a nitrogen‐filled environment. The hydrolysate of each sample (100 μL) was evaporated using a Termovap Sample Concentrator, cooled to room temperature, and diluted to 60 mL with water. The samples were derivatized using an AA determination kit (MSLAB‐45 + AA, MS Lab, Beijing, China) to determine their compositions. Thereafter, 50 μL of derivatized solution was separated for HPLC‐MS/MS quantification. An MSLab 45 + AA‐C18 Column (5 μm, 4.6 mm l.D. × 150 mm; MS Lab, Beijing, China) was employed for chromatographic separation at 50°C using an injection volume of 3 μL. Eluent A consisted of ultrapure water with 0.1% formic acid, while eluent B comprised acetonitrile with 0.1% formic acid. The flow rate was 1.0 mL/min. The gradient elution procedure is shown in Table 1. The AA concentration was estimated from the standard curve of the mixed AA standards (MS Lab, Beijing, China).

**TABLE 1:** The gradient elution procedure, including sample analysis in MRM Scan mode, positive ionization, IS: +5500 V, GS1: 55 psi, GS2: 60 psi, CAD: Medium, TEM: 500°C, CUR: 20 psi, CXP: 2.0, and EP: 10.

Gradient	1	2	3	4	5	6	7	8
Retention	0.01	6.0	6.10	10.0	10.1	12.5	12.6	15.0
%A	12	17	24	27	97	97	12	12
%B	88	83	76	73	3	3	88	88

The AA scores of the individual essential AAs were calculated according to FAO/WHO/UNU scoring patterns for adults using the following equation (FAO, 2013):

(1) AAScore=mgofAAin1gof test protein/mgof the sameAAin1gof reference protein

### 2.3 In vitro gastrointestinal digestion

The in vitro gastrointestinal digestion system was based on the INFOGEST 2.0 protocol with slight modifications (Brodkorb et al., 2019). Briefly, the digesta containing 250 mg of protein were diluted to 10 mL with water (and 10 mL water alone for blank control), after which 10 mL (1:1, *v*:*v*) of simulated gastric fluid (SGF) containing pepsin (2000 U/mL in total digesta) was added and shaken continuously for 120 min at pH 2.0. Next, 20 mL (1:1, *v*:*v*) of simulated intestinal fluid (SIF), consisting of porcine pancreatic trypsin (100 U/mL in total digesta), lipase (250 U/mL in total digesta), and porcine bile (10 mM in total digesta), was incubated for 120 min at pH 7.0. The digestion simulative phases were incubated at 37°C in a water bath at 200 r/m. During gastric digestion, 500 μL of digesta were collected at 0, 60, and 120 min during gastric digestion (G0, G60, and G120), and 60 and 120 min during intestinal digestion (I60 and I120), respectively. At once, the protein digestion was terminated by a boiling water bath for 10 min. The samples were stored at −20°C until further use, and all procedures were repeated three times.

### 2.4 Determination of particle size distribution

The particle size distribution analysis before and during the simulated digestion was measured based on a previously described method (Li & Fan, 2020) using a laser diffraction particle analyzer (Microtrac S3500, Microtrac Instruments Ltd., USA). All the digesta samples were immediately placed in an ice bath for detection and analyzed in triplicate.

### 2.5 Determination of the total free amino group

The total free amino group content in the supernatant digesta was determined based on a previously described ninhydrin colorimetric method with slight modifications (Qiu et al., 2023). Here, 500 μL of the digesta obtained in Section 2.3 was diluted to 1.5 mL and centrifuged at 8000 × *g* for 5 min. The supernatant (500 μL) was incubated for 15 min in a boiling water bath with phosphate buffer (pH 8.0, 50 μL) and ninhydrin reagent (50 μL). The reaction solution was immediately cooled to room temperature, and the absorbance was measured at 570 nm. The free amino group concentration was estimated against the L‐leucine standard curve, expressed as L‐leucine equivalent. All samples were analyzed in triplicate.

### 2.6 Sodium dodecyl sulfate‐polyacrylamide gel electrophoresis (SDS‐PAGE)

The digesta were exposed to SDS‐PAGE gels based on the previously described method with slight modifications (Hodgkinson et al., 2018; Ma et al., 2023). Briefly, the digesta were mixed with 5 × protein loading buffer (Shanghai Yamei Biomedical Technology, Shanghai, China) at a 4:1 (*v*:*v*) ratio and heated at 100°C for 5–10 min. The mixture was cooled to room temperature and centrifuged at 14,111 × *g* for 5 min. Next, 20 μL of the supernatant digesta was separated with 12% Super‐PAGE™ gel (Shanghai Yamei Biomedical Technology, Shanghai, China) and processed at a constant voltage of 150 V for 55 min. Protein markers from 11 kDa to 180 kDa were used to determine the molecular weight distribution, and the strips were measured with Coomassie bright blue staining method.

### 2.7 Gel permeation chromatography (GPC)

The molecular weight distribution of small proteins was determined by GPC using a Waters HPLC system (equipped with a 2487 UV detector; Waters Corporation, Milford, MA, USA). The digesta was diluted to 1 mg/mL with the eluent, which consisted of acetonitrile–water–trifluoroacetic acid at a 20:80:0.1 (*v*:*v*:*v*) ratio. After being centrifuged (8000 × *g*, 5 min), the supernatant solution was passed through a 0.22‐μm tetrafluoroethylene filter membrane (Jinteng Experimental Equipment, Tianjin, China). A TSKgel UP‐SW2000 column (2 μm, 4.6 mm l.D. × 30 cm; Tosoh Corporation, Tokyo, Japan) was employed for chromatographic separation at 25°C using an injection volume of 20 μL. The flow rate was 1.0 mL/min, and samples were detected at 220 nm by an ultraviolet detector. Cytochrome C (12,384 Da), porcine pancreatic insulin (5778 Da), bacitracin (1423 Da), glycyl‐glycyl‐tyrosyl‐arginine (451 Da), and triglycine (189 Da) were used as the standards to estimate the correction curve. All samples were analyzed in triplicate.

### 2.8 Determination of FAA profiles

The FAA profiles were determined using HPLC‐MS/MS. Two aliquots of each digesta were diluted with ultrapure water at a 1:3 (*v*:*v*) ratio, and then all the dilutions were derivatized and determined as described in Section 2.2.

### 2.9 Statistical analysis

The data were analyzed using SPSS version 25.0 (SPSS Inc., Chicago, IL., USA) and subjected to one‐way analysis of variance (ANOVA). The means were compared using Duncan's multiple range test at a 95% confidence level to determine significant differences between the samples (*p* < .05). Additionally, the molecular weight distribution of small proteins was processed by GPC software (Waters Corporation, Milford, MA, USA). All experiments were performed in duplicate, and the data are presented as means ± SEM.

## 3 RESULTS AND DISCUSSION

### 3.1 Protein content, total AA content, and AA score analysis of different samples

To characterize the nitrogen fraction of the Multi‐PBMA, soy and bovine milk, nitrogen content, and total AA profiles were determined using the Kjeldahl method and HPLC‐MS/MS, respectively. AA contents were expressed as percentages of crude protein (CP) content to rule out the effects of different protein levels among samples (Table 2). As shown, Multi‐PBMA had a higher proportion of essential AAs (36.45%) than soy milk (29.91%). Furthermore, the Multi‐PBMA was richer in histidine, isoleucine, aromatic AAs (AAAs, phenylalanine + tyrosine), alanine, cysteine, glycine, and serine, as well as less in sulfur AAs (SAAs, methionine + cysteine) than the bovine milk that was regarded as the high‐quality protein source.

**TABLE 2:** The CP and AA profiles in the Multi‐PBMA, soy, and bovine milk.

	Multi‐PBMA	Soy milk	Bovine milk
Crude protein (CP, %, w/w)	3.01	5.92	3.52
Essential AAs (% CP)			
Histidine	2.60	2.00	2.08
Isoleucine	4.31	3.33	4.03
Leucine	7.66	5.95	7.93
Lysine	6.13	5.35	7.90
Methionine	1.00	0.85	2.16
Phenylalanine	4.38	3.43	3.35
Threonine	3.95	3.16	3.79
Tryptophan	1.33	1.69	1.70
Valine	5.43	4.18	5.78
Total essential AAs	36.45	29.91	38.50
Non‐essential AAs (% CP)			
Arginine	7.43	8.11	3.41
Alanine	4.47	4.39	3.41
Aspartic acid	8.50	12.50	7.95
Cysteine	0.46	0.31	0.19
Glutamic acid	19.24	20.10	21.31
Glycine	3.85	2.89	1.47
Proline	4.71	3.91	7.32
Serine	4.18	3.19	3.57
Tyrosine	1.99	1.72	1.94
Total non‐essential AAs	54.83	57.12	50.57

*Note*: The AA content was calculated as g/100 g (%) CP. The values represent the average of two analytical replicates.

To further evaluate protein quality, the AA score based on the amount of the first limiting AA was calculated with the FAO/WHO/UNU reference AA scoring patterns for adults (Table 3). The first limiting amino acid of the Multi‐PBMA was SAAs with a score of 0.60, slightly higher than that of the soy milk (with a score of 0.54), while that of the bovine milk was AAAs (with a score of 0.88). These results indicated that the Multi‐PBMA had a higher protein quality and better‐balanced AA profiles by comprising various grains (soybeans and cereals).

**TABLE 3:** The AA scores of the Multi‐PBMA, soy, and bovine milk.

AA	FAO/WHO/UNU scoring pattern mg/g protein requirement	Multi‐PBMA	Soy milk	Bovine milk
Histidine	15	1.73	1.33	1.39
Isoleucine	30	1.44	1.11	1.34
Leucine	59	1.30	1.01	1.34
Lysine	45	1.36	1.19	1.76
SAAs	22	**0.60**	**0.54**	1.11
AAAs	38	1.15	0.90	**0.88**
Threonine	23	1.72	1.38	1.65
Tryptophan	6	2.21	2.82	2.84
Valine	39	1.39	1.07	1.48

*Note*: The values in bold indicate the first limiting AA. SAA, sulfur AAs (including methionine and cysteine); AAA, aromatic AAs (including phenylalanine and tyrosine).

### 3.2 Proteolysis during in vitro digestion

**FIGURE 1:**
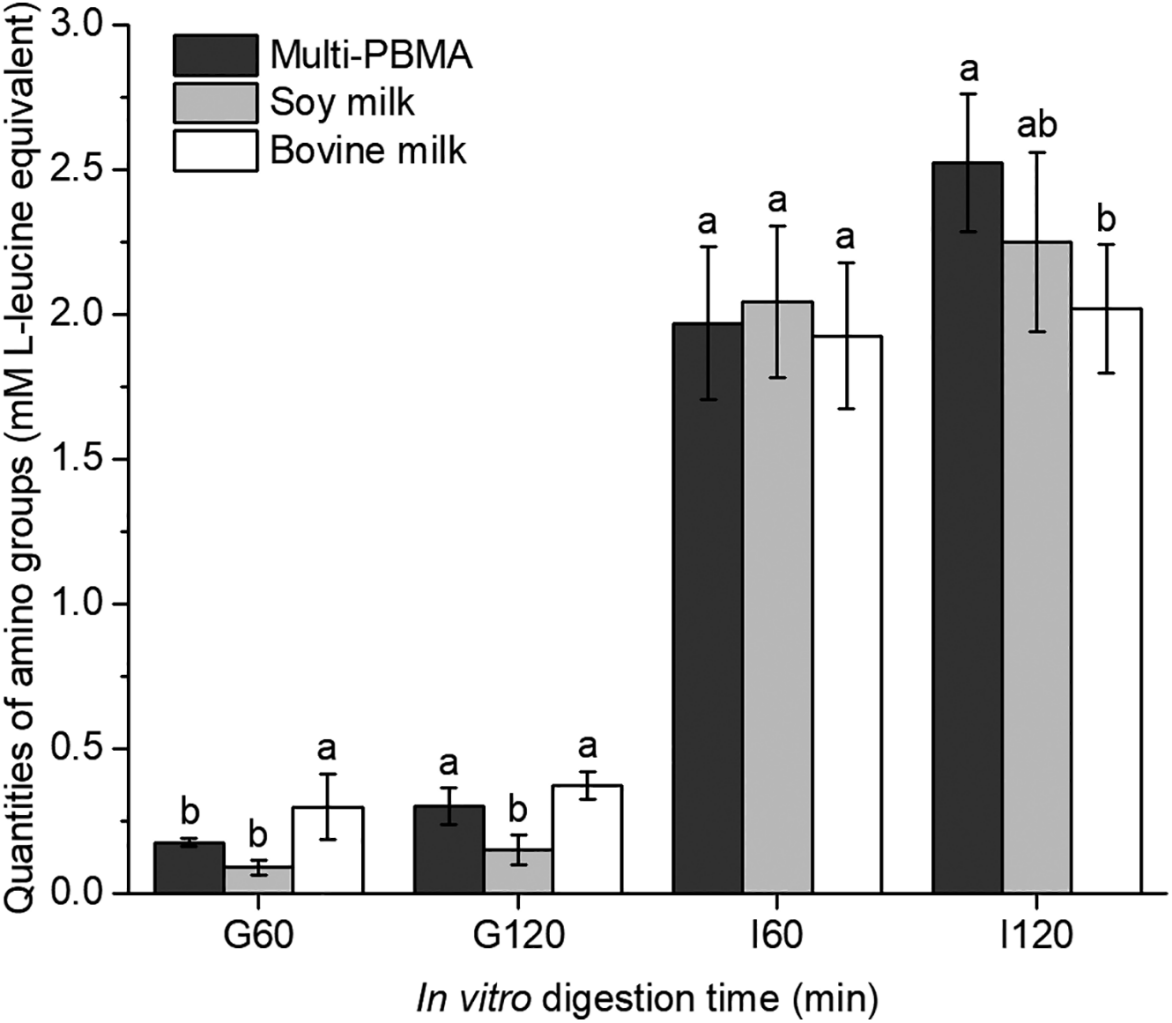
The quantities of amino groups (expressed as L‐leucine equivalent) during the in vitro gastrointestinal digestion of Multi‐PBMA, soy, and bovine milk. G: gastric digestion. I: intestinal digestion. The numbers following G and I represent the digestion time. Different letters indicate significant differences between the samples at the same time point (*p* < .05). The data and error bars represent the means and SEM, *n* = 3.

The proteolytic degree is one of the prime dimensions of protein gastrointestinal behavior. To evaluate the proteolytic degree during in vitro gastrointestinal digestion availability, the quantities of amino groups in digesta were measured using the ninhydrin colorimetric method and expressed as L‐leucine equivalent. A higher proteolytic degree in the Multi‐PBMA during in vitro digestion is demonstrated in Figure 1. As shown in gastric digestion (G60 and G120), proteolysis under pepsin was limited. The proteolytic degree of the Multi‐PBMA was higher at the end (G120) than that of the soy milk (*p* < .05), while there was no significant difference with the bovine milk (*p* > .05). As shown in intestinal digestion (I60 and I120), the proteolytic degree subsequently increased at the beginning, indicating that the remaining proteins in the gastric chyme continued to hydrolyze rapidly. Generally, the proteolysis of the Multi‐PBMA increased with intestinal digestion, but fewer protein hydrolytic changes occurred in the bovine milk. At the end of digestion (I120), the Multi‐PBMA displayed the highest proteolytic degree (2.52 ± 0.24 mM), followed by soy (2.25 ± 0.31 mM) and bovine milk (2.02 ± 0.22 mM). The significant differences between the Multi‐PBMA and the bovine milk were apparent (*p* < .05), while there was no difference between the Multi‐PBMA and soy milk (*p* > .05). The relevant statistical output was attached in the Table S1.

During gastrointestinal digestion, limited proteolysis at the gastric phase and swift proteolysis at the intestinal phase demonstrated that pepsin and pancreatin could catalyze the generation of short peptide and FAA from proteins, respectively (Wardlaw, 1999). The higher degree of proteolysis in the Multi‐PBMA was probably attributed to germinated soybeans with intrinsic protein hydrolase activation and fermented cereals with cotyledonary cell integrity disruption, increasing the in vitro protein digestibility. Previous studies also highlighted the ability of germination and fermentation to improve plant protein digestibility (Bera et al., 2023). Furthermore, the complex proteins stored in plant cells were partially degraded into more structurally simple and soluble molecules via preprocessing techniques, making them more susceptible to protease attack (Yousif & El Tinay, 2001). Therefore, the Multi‐PBMA containing germinated soybeans and fermented cereals possesses an advantage in protein hydrolysis over soy and bovine milk as a potential protein food that can be absorbed more easily by the human body.

### 3.3 Characteristics of in vitro digestion

#### 3.3.1 Particle size distribution during protein digestion

**FIGURE 2:**
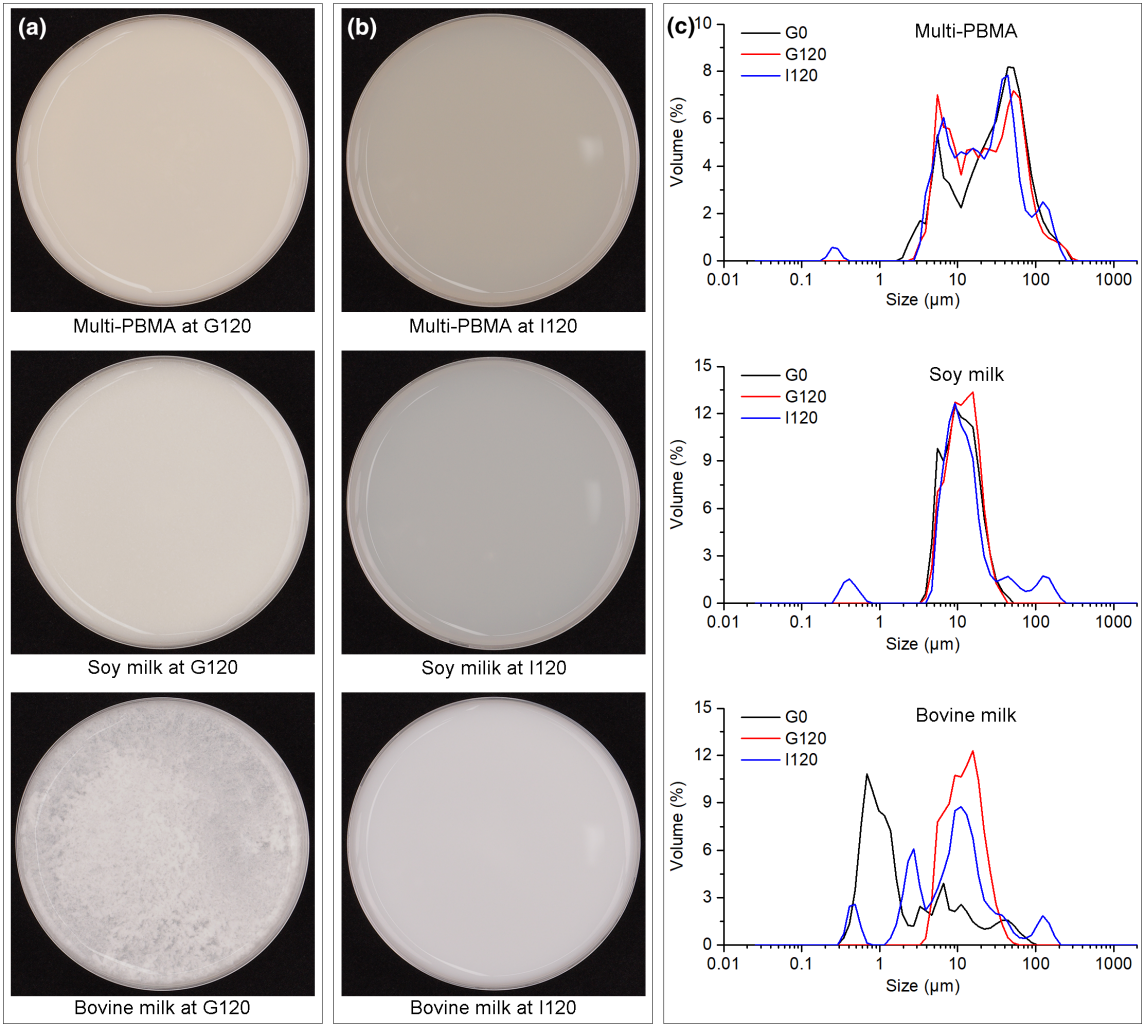
The images of the digesta collected at 120 min of gastric (a) and intestinal (b) digestion, and the particle size distribution (c) during digestion (plotted according to the triplicate averages).

The digestion behavior of different proteins could be affected evidently by the acidic gastric environment or complex digestive matrix. Different protein digestible responses of the Multi‐PBMA and other two kinds of milks were observed when they were exposed to SGF. As shown in Figure 2a, the Multi‐PBMA displayed no visible coagulation, the same as the soy milk, while the bovine milk exhibited distinctive coagulation characteristics throughout gastric digestion (G120). This phenomenon could be attributed to the low pH and pepsin function in the SGF, promoting curd formation in the bovine milk with a high level of casein, which was consistent with previous studies (H. Zhang et al., 2023). Several studies reported that the soft and fragile curd formed in protein coagulation could contribute to a slow protein transit through the stomach and the controlled release of AAs into the blood during intestinal digestion, while the hard curd formation could be associated with gastrointestinal symptoms (Huppertz & Chia, 2021). As shown in Figure 2b, the gastric curd in different digesta was mostly invisible when entering intestinal digestion, which could be due to neutral SIF conditions that increased protein coagulation solubility and prevented obvious curd formation during intestinal digestion under pancreatin function, especially in bovine milk digesta (H. Zhang et al., 2023). Consistently, plant proteins are demonstrated to be more gut‐friendly relative to animal proteins that have the possibility to cause a delay in nutrient emptying, digestion, and absorption kinetics (Mulet‐Cabero et al., 2020).

In order to further characterize the morphological differences of digesta, particle distribution analyses of digesta at G0, G120, and I120 were measured using a particle size analyzer. As shown in Figure 2c, the Multi‐PBMA displayed the most extensive particle distribution, which may be related to fiber, lipid, and polysaccharide from preprocessed various grain ingredients. The particle size distribution of the soy and bovine milk yielded a sharp single peak, indicating a single or simple composition (Figure 2c). From the curves at G0, G120, and I120 in Figure 2c, the particle size distribution in the Multi‐PBMA digesta was not as obvious as that in the soy milk digesta. In contrast, as shown in Figure 2c, the particle sizes in the bovine milk digesta increased dramatically during gastric digestion (from G0 to G120) and then decreased during intestinal digestion (from G120 to I120), indicating the protein (e.g. casein) coagulation in SGF because of protein cross‐linking. Interestingly, the Multi‐PBMA digesta presented a far wider range of particle size distribution with invisible protein coagulation, while the bovine milk exhibited distinct coagulation with a narrower distribution of particle sizes (Figure 2). These results suggested that the different particle distribution may not be directly related to the protein digestibility in the Multi‐PBMA as well as the soy milk digesta compared to the bovine milk.

With consideration of the results in Figure 1, the larger particle size distribution in the Multi‐PBMA digesta rarely seems to affect the proteolytic degree, which differs from the previous study showing that a particle size decrease would be beneficial for better in vitro digestion (Capuano & Pellegrini, 2019). This may be due to the differences in the level of particle size distribution, and the previous study mainly focused on nutrient release rather than protein digestibility. Several studies have demonstrated that the curd formation was not detected in human milk because of the low casein content while a large particle size distribution existed during *in vitro* digestion (Zou et al., 2022). Therefore, we speculate that Multi‐PBMA containing various preprocessed grain ingredients may be equipped with a similar microstructure as human milk during the whole digestion process, resulting from the complicated protein–lipid droplet, which still needs further research.

#### 3.3.2 The molecular weight distribution of the proteins and peptides

**FIGURE 3:**
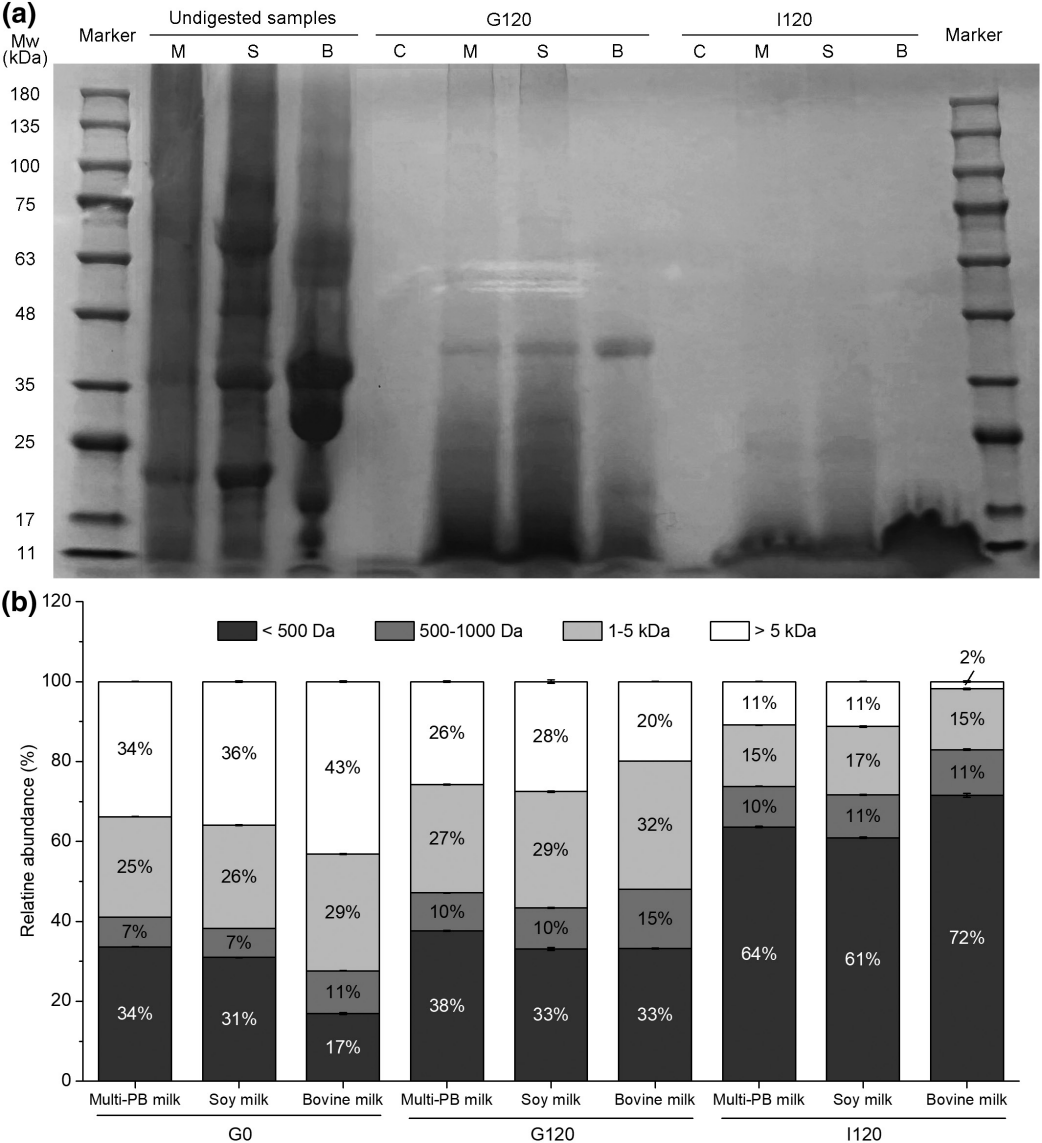
The SDS‐PAGE protein profiles (a) and molecular weight distribution of proteins before and after digestion (b). G: gastric digestion. I: intestinal digestion. The numbers following G and I represent digestion time. M: Multi‐PBMA milk. S: soy milk. B: bovine milk. C: blank control. The data and error bars represent the means and SEM, *n* = 3.

The molecular weight distribution of the proteins and peptides was determined using SDS‐PAGE and GPC to discuss the protein digestion characteristics, as shown in Figure 3. The proteins and peptides in digesta from different samples were characterized at 11–180 kDa (Figure 3a). As shown in Figure 3a, three protein bands in the undigested Multi‐PBMA and soy milk were observed, with molecular weights of 17–25, 35–48, and 63–75 kDa, respectively. This result was consistent with the previous study, which found that undigested soy‐based foods probably had the three prominent protein bands corresponding to lipoxygenase isoforms 1, 2, and 3, the α, α', and β subunits of β‐conglycinin, and acidic and basic glycinin subunits (Liu et al., 2019). And the undigested bovine milk presented visible bands in the range of 25–35, 35–48, and 48–63 kDa, which may be α_s_, β, κ‐casein, β‐lactoglobulin, and α‐lactalbumin reported from several studies (Nguyen et al., 2020). As shown in Figure 3a, after gastric digestion (G120), the protein band of 35–48 kDa in three digesta was still evident while almost disappearing after intestinal digestion (I120). The findings indicated the gradual release of trapped soluble proteins from the protein–lipid–polysaccharide complex during digestion (Liu et al., 2019). However, there were no obvious protein bands observed in the intestinal digesta, suggesting that protein digestion and degradation into smaller peptides <17 kDa could not be identified successfully.

Accordingly, GPC was used to further characterize the smaller soluble peptides. The peptide percentages in the Multi‐PBMA, soy, and bovine milk before and after gastric and intestinal digestion (G0, G120, and I120) are presented in Figure 3b. Generally, both in the process of gastric and intestinal digestion, the peptide percentage (< 1 kDa) increased constantly in all samples, and the peptide percentage (>5 kDa) decreased gradually, indicating the degradation of larger‐molecular‐weight proteins into lower‐molecular‐weight proteins (i.e. peptides) due to optimal protease utilization in the *in vitro* digestion system. At the beginning (G0), the abundance of peptides <500 Da was the highest (34%), while other fractions were the lowest in the Multi‐PBMA. Furthermore, the increase in the abundance of peptides <500 Da in the Multi‐PBMA was comparable to that in soy milk, but less than that in bovine milk during the whole digestion process. Compared with the soy milk, the higher level of <500 Da peptides and the lower level of >5 kDa peptides in the undigested Multi‐PBMA proved that the low‐molecular‐weight proteins could be enriched via the various grains preprocessed by germination and fermentation techniques. Given the peptide percentage (<500 Da) in the Multi‐PBMA and bovine milk before (G0) and after digestion (G120 and I120), it was indicated that the animal‐based food has a larger but better digestible protein structure than the plant‐based food. Overall, during the digestion process, the molecular weight distribution of smaller proteins (i.e. peptide) in the Multi‐PBMA was consistently higher than that in the soy milk, which could be due to the benefit of germination and fermentation techniques on improving plant protein digestibility quality (Bera et al., 2023).

#### 3.3.3 FAA profiles during in vitro digestion

**FIGURE 4:**
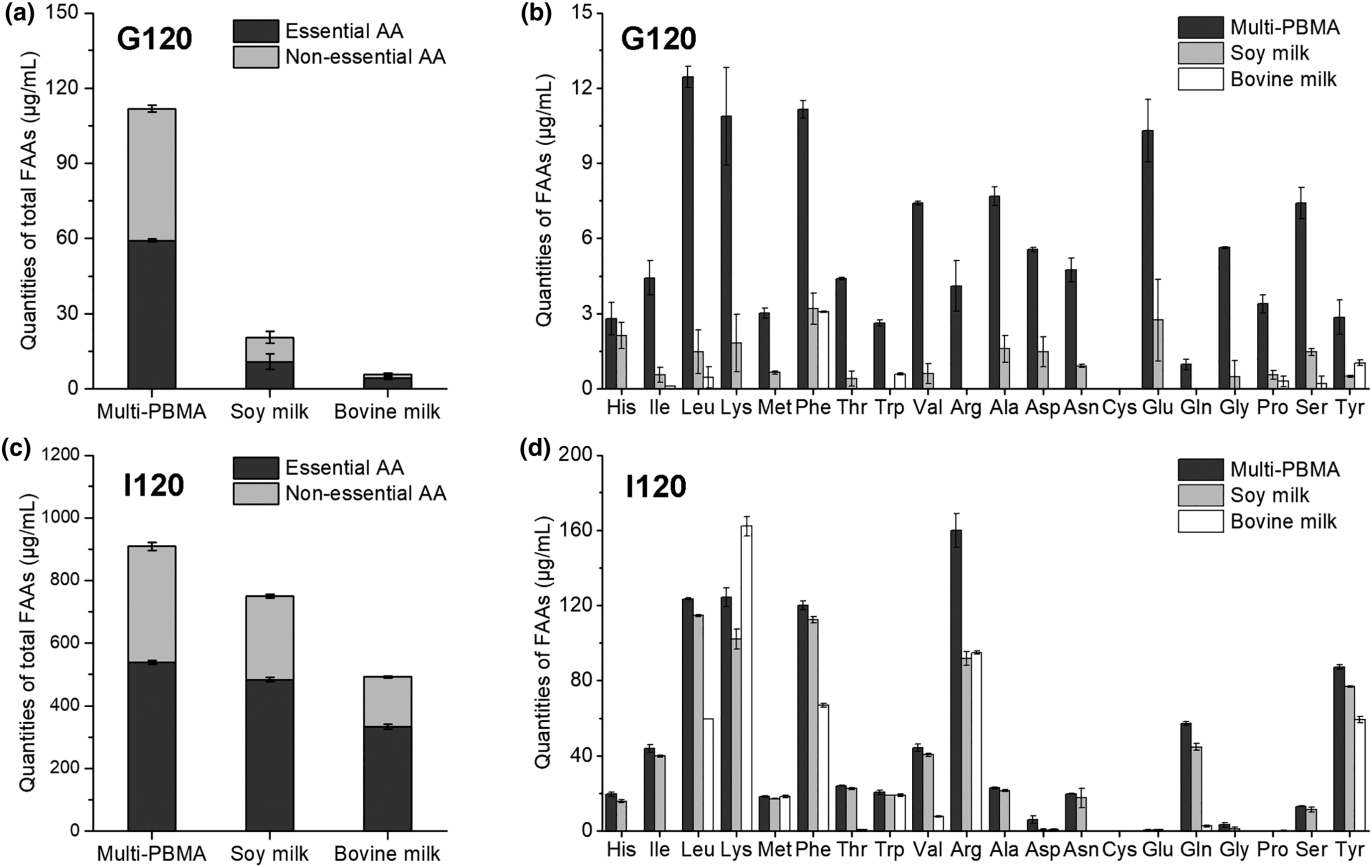
The profile of total FAAs after gastric (a) and intestinal (b) digestion. The FAA profiles after gastric (c) and intestinal (d) digestion. The data and error bars represent the means and SEM, *n* = 3.

The FAA profiles after digestion in three digesta were determined by HPLC‐MS/MS for evaluating their potential nutritional value. Notably, the total FAAs and most of the single AAs (e.g. essential AAs other than lysine) in the Multi‐PBMA were much higher than those in the other samples during the whole digestion process (Figure 4), which could not be discriminated using GPC at 220 nm because of the size exclusion separation mechanism. As shown in Figure 4a,c, the Multi‐PBMA had a prominent advantage in terms of essential, non‐essential, and total FAA contents in contrast with soy and bovine milk, especially during the gastric digestion phase. On the basis of the results of peptide molecular weight distribution (Figure 3b), since the Multi‐PBMA consisted of smaller proteins, they were more likely to be directly hydrolyzed into monomolecular AAs than low‐molecule proteins (i.e. peptides). However, proteins in the bovine milk were likelier to be digested by pepsin into peptide segments (Nguyen et al., 2020). This could be attributed to the different microstructures and kinetics behind protein enzymatic proteolysis in plant and animal proteins. Studies have reported that the peptic hydrolysis of native globular proteins had two extreme types of reactions: one was that proteins were hydrolyzed one after another in a sequence with undetectable intermediate products; the other was that protein hydrolysis started quickly at an initial stage and then it slowed down subsequently with a wide range of peptide products generated; above all, most proteases could participate in and be active within these two reactions (Luo et al., 2015). As shown in Table 2, the Multi‐PBMA had the highest AAAs with 6.37% CP (and 5.15% and 5.29% CP in the soy and bovine milk, respectively), especially phenylalanine with 4.38% CP (and 3.43% and 3.35% CP in the soy and bovine milk, respectively). It was speculated that protease function in different types, as mentioned above, might work according to the AA fraction and protein microarchitecture in different milk samples. For instance, pepsin tended to prefer action sites with hydrophobic residues, especially AAA residues including phenylalanine and tyrosine (Luo et al., 2015). However, whether the high levels of phenylalanine and AAAs could affect the hydrolysis types of pepsin was worthy of further investigation in the future.

Besides, a comparison of total FAAs between the Multi‐PBMA and the soy milk (Figure 4a,c) indicated that the higher FAA level was possibly due to the adequate protein hydrolysis during germination, which increased the susceptibility to digestive enzymatic attack (Albarracin et al., 2019). As reported, germination plays an important role in protein pre‐digestion into smaller peptides, increasing protein digestibility in the gastric digestion phase (Bautista‐Exposito et al., 2021). And fermentation disrupted cell integrity and possibly promoted protein migration or degradation, increasing the FAA content during gastrointestinal digestion (Sun et al., 2023). In addition, the disparity of total FAAs between the Multi‐PBMA and the bovine milk decreased significantly (Figure 4a,c), indicating that proteins in the Multi‐PBMA were continuously digested during gastrointestinal digestion while those in the bovine milk were hydrolyzed mainly during intestine digestion. These findings suggested that the Multi‐PBMA containing the germinated soybeans and fermented cereals still displayed a distinct advantage during the whole digestion phase.

Furthermore, each FAA profile in three milk samples during gastrointestinal digestion was provided, as shown in Figure 4b,d. No cysteine was released in any of the digesta during in vitro digestion. All other FAAs were generated in the Multi‐PBMA at the end of gastric digestion (G120), but some of them were presented in the soy and bovine milk merely (Figure 4b). In detail, no tryptophan, arginine, and glutamine were generated in the soy milk during the gastric digestion phase, while the bovine milk contained isoleucine, leucine, and tryptophan as essential AAs and proline, serine, and tyrosine as non‐essential AAs. Additionally, as shown in Figure 4d, leucine, lysine, and phenylalanine were the most abundant essential FAAs in the Multi‐PBMA, as well as in the soy milk, during the intestinal digestion phase, while the intestinal digesta of the bovine milk were rich in lysine, phenylalanine, and leucine. In the intestinal digesta of the Multi‐PBMA and the soy milk, arginine and tyrosine were the most abundant non‐essential AAs in all digests, followed by the total amount of glutamic acid and glutamine. The results were consistent with previous studies, showing that leucine, lycine, phenylalanine, and arginine represented the most abundant FAAs in the legume protein isolates, casein, and whey proteins after gastrointestinal digestion (Santos‐Hernandez et al., 2020).

These results proposed a question about the relationship between the proteolysis degree and the protein/peptide molecular weight distribution. In the Multi‐PBMA, the proteins seem to be directly hydrolyzed into large amounts of FAAs, to a greater extent than the commercial soy milk, which presented a potential ability for rapid absorption and utilization in the human body. And it was stated that incomplete protein digestion and peptide retention may affect the AA utilization efficiency in protein synthesis for the human body (Zhang et al., 2022). In a way, protein‐incomplete digestion could keep bioactive fragments intact, allowing intestinal absorption or the enhancement of organ functionality in the intestinal tract (Zhang et al., 2022). In addition, as shown in Figure 4b, except for the lower lysine content in the Multi‐PBMA than that in the bovine milk after intestinal digestion, other FAAs were superior to that in the soy and bovine milk. Besides proteins, there were additional benefits in unsaturated fatty acids, dietary fiber, and some secondary plant metabolites provided by the PB milk alternatives (Xiao et al., 2023). Therefore, the results indicated that the Multi‐PBMA could provide excellent advantages in terms of protein digestibility behavior and nutritional properties compared with soy and bovine milk, exhibiting a detailed insight into protein digestibility properties in complex plant proteins.

## 4 CONCLUSIONS

This study provides a novel Multi‐PBMA containing various germinated soybeans and fermented cereals and evaluates its nutritional value based on protein digestibility, coagulation characteristics, particle size distribution, molecular weight distribution, and FAA profile via an in vitro gastrointestinal digestion system. The data showed that the Multi‐PBMA had a higher proportion of essential AAs and AAAs, as well as a better AA score with SAAs as the first limiting AA, than the soy milk. Overall, the Multi‐PBMA displayed better performances in protein digestibility behavior and better balanced FAA profiles during digestion, compared with commercial soy and bovine milk. Furthermore, Multi‐PBMA displayed a protein digestible response with no apparent gastric coagulation, which may be more gut‐friendly than bovine milk. The particle size analysis of the Multi‐PBMA showed that its extensive particle distribution had little effect on its protein digestibility, suggesting that similar microstructures (e.g. protein–lipid droplets) exist in human milk. Combining the results of proteolytic degree, molecular weight distribution, and composition analysis of FAA profiles, we suspected that the proteins in the Multi‐PBMA were inclined to hydrolyze directly into monomolecular AAs rather than low‐molecular‐weight proteins (i.e. peptides) as intermediate products, owing to a different type of protein hydrolysis probably from that in bovine milk.

In conclusion, this study provides one new insight into the comprehensive understanding of single or complex plant protein and animal protein digestion behaviors. Additionally, germination and fermentation as plant preprocessing techniques could be effective in improving the nutritional quality of plant‐based products. The higher proteolytic degree and better balanced AA profiles in the Multi‐PBMA under in vitro digestion suggest that it would be a promising industrial plant‐based product with excellent nutritional value. Further research should focus on the microkinetics of plant protein hydrolysis in PB milk alternatives and their multi‐scale structures (e.g. protein–lipid droplets as in human milk) under in vitro/in vivo digestion condition. And the different digestive behaviors between plant protein and other animal proteins, such as goat, camel, and donkey milk, are also worth further investigation, which will provide the basis for more proof‐of‐concept works on the health improvement function of plant proteins. In pace with intensive study and expanded consumption, if the demand for ‘plant‐based’ will increase significantly in the future, the plant‐based industry should really deal with the deforestation plague.

## AUTHOR CONTRIBUTIONS

**Xue Wang:** Conceptualization (equal); resources (equal). **Lu Zhang:** Conceptualization (equal); resources (equal); writing – review and editing (equal). **Mohan Wang:** Methodology (equal); validation (equal); writing – original draft (equal). **Hongjiang Ma:** Methodology (equal). **Shiwei Liu:** Validation (equal). **Meng Wang:** Validation (equal); writing – original draft (equal). **Youqiang Yu:** Validation (equal); writing – original draft (equal). **Guoyu Liu:** Investigation (equal). **Qiuge Cao:** Data curation (equal). **Xi Wang:** Data curation (equal). **Xishan Ma:** Data curation (equal). **Peng Yuan:** Formal analysis (equal). **Jia Liu:** Formal analysis (equal). **Yongjiu Zhang:** Funding acquisition (equal); resources (equal); supervision (equal). **Shenglin Duan:** Project administration (equal); resources (equal); supervision (equal).

## CONFLICT OF INTEREST STATEMENT

The authors declare no conflicts of interest.

## Supporting information


Table S1

## Data Availability

The data that support the findings of this study are available on request from the corresponding author. The data are not publicly available due to privacy or ethical restrictions.
