# Supplementary material for: A novel multiple plant‐based milk alternative containing various preprocessed grains achieves better performance in protein digestibility and free amino acid profile via in vitro gastrointestinal digestion analysis
Source: Food Sci Nutr. 2024 Jun 26;12(9):6637–47. doi: 10.1002/fsn3.4177 (PMC11561810; doi:10.1002/fsn3.4177)
Supplement: Supplementary file 1 — Table S1 [file FSN3-12-6637-s001.docx]

**Supplementary material**

**Supplementary Table 1**

The quantities of amino groups (expressed as L-leucine equivalent) during the *in vitro* gastrointestinal digestion of Multi-PBMA, soy, and bovine milk.

|  | G60 | G120 | I60 | I120 |
| --- | --- | --- | --- | --- |
| Multi-PBMA | 0.18 ± 0.01^b^ | 0.30 ± 0.06^a^ | 1.97 ± 0.26^a^ | 2.52 ± 0.24^a^ |
| Soy milk | 0.09 ± 0.03^b^ | 0.15 ± 0.05^b^ | 2.04 ± 0.26^a^ | 2.25 ± 0.31^ab^ |
| Bovine milk | 0.30 ± 0.11^a^ | 0.37 ± 0.05^a^ | 1.92 ± 0.25^a^ | 2.02 ± 0.22^b^ |

*Notes:* G: gastric digestion. I: intestinal digestion. The numbers following G and I represent the digestion time. Different letters in the same column indicate significant differences between the samples at the same time point (*P* < 0.05). The data and error bars represent the means and SEM, *n* = 3.
